# EMBRACE intervention to improve the continuum of care in maternal and newborn health in Ghana: The RE-AIM framework-based evaluation

**DOI:** 10.7189/jogh.11.04017

**Published:** 2021-03-27

**Authors:** Kimiyo Kikuchi, Margaret Gyapong, Akira Shibanuma, Evelyn Ansah, Sumiyo Okawa, Sheila Addei, Keiko Nanishi, Charlotte Tawiah, Junko Yasuoka, Francis Yeji, Abraham Oduro, Seth Owusu-Agyei, Gloria Quansah-Asare, Abraham Hodgson, Masamine Jimba, the Ghana EMBRACE Implementation Research Team

**Affiliations:** 1 Department of Community and Global Health, Graduate School of Medicine, The University of Tokyo, Tokyo, Japan; 2 Department of Health Sciences, Faculty of Medical Sciences, Kyushu University, Fukuoka, Japan; 3 Institute for Health Research, University of Health and Allied Sciences, Volta, Ghana; 4 Dodowa Health Research Centre, Dodowa, Greater Accra, Ghana; 5 Research and Development Division, Ghana Health Service, Accra, Ghana; 6 Cancer Control Center, Osaka International Cancer Institute, Osaka, Japan; 7 Office of International Academic Affairs, Graduate School of Medicine and Faculty of Medicine, The University of Tokyo, Tokyo, Japan; 8 Kintampo Health Research Centre, Kintampo, Brong-Ahafo, Ghana; 9 Research and Education Center for Prevention of Global Infectious Diseases of Animals, Tokyo University of Agriculture and Technology, Tokyo, Japan; 10 Navrongo Health Research Centre, Navrongo, Upper East, Ghana; 11 Ghana Health Service, Accra, Ghana

## Abstract

**Background:**

Improving maternal and newborn health remains one of the most critical public health challenges, particularly in low- and lower-middle-income countries. To overcome this challenge, interventions to improve the continuum of care based on real-world settings need to be provided. The Ghana Ensure Mothers and Babies Regular Access to Care (EMBRACE) Implementation Research Team conducted a unique intervention program involving over 21 000 women to improve the continuum of care, thereby demonstrating an intervention program’s effectiveness in a real-world setting. This study evaluates the implementation process of the EMBRACE intervention program based on the RE-AIM framework.

**Methods:**

A cluster-randomized controlled trial was conducted in 32 sub-district-based clusters in Ghana. Interventions comprised of four components, and to evaluate the implementation process, we conducted baseline and endline questionnaire surveys for women who gave birth and lived in the study site. The key informant interviews of health workers and intervention monitoring were conducted at the health facilities in the intervention area. The data were analyzed using 34 components of the RE-AIM framework and classified under five general criteria (Reach, Effectiveness, Adoption, Implementation, and Maintenance).

**Results:**

In total, 1480 and 1490 women participated in the baseline and endline questionnaire survey, respectively. In the intervention area, 83.8% of women participated (reach). The completion rate of the continuum of care increased from 7.5% to 47.1%. Newborns who had danger signs immediately after birth decreased after the intervention (relative risk = 0.82, 95% confidence interval = 0.68-0.99) (effectiveness). In the intervention area, 94% of all health facilities participated. Mothers willing to use their continuum of care cards in future pregnancies reached 87% (adoption). Supervision and manual use resolved the logistical and human resource challenges identified initially (implementation). The government included the continuum of care measures in their routine program and developed a new Maternal and Child Health Record Book, which was successfully disseminated nationwide (maintenance).

**Conclusions:**

Following the RE-AIM framework evaluation, the EMBRACE intervention program was considered effective and as having great potential for scaling across in real-world settings, especially where the continuum of care needs to be improved.

**Trial registration:**

ISRCTN 90618993.

Globally, maternal and newborn health care access has improved within the framework of the Millennium Development Goals [1], but progress remains slow in many low- and lower-middle-income countries, such as Ghana [2]. Consequently, the Sustainable Development Goals aim at reducing maternal, newborn, and child mortality among other targets [3].

The continuum of care (CoC) has recently been advocated as a means of improving mothers’ and newborns’ health. The theoretical consensus around the definition of CoC looks at it as an approach that allows mothers and newborns to continuously receive care from skilled care providers [4-6]. A meta-analysis has suggested that CoC can improve mothers’ and newborns’ health [7,8]. Although CoC is considered an effective element of public health, it is rarely practiced in low- and lower-middle-income countries, and interventions are required to promote its use [9].

Ghana, a lower-middle-income country, still faces challenges regarding access to maternal and newborn health care services. In 2014, the rate of attending four or more antenatal care (ANC) sessions in the country was high (87.3%); however, the rate of postnatal care (PNC) within the first two days after birth was low at 22.8% [10]. Additionally, health care access has been considerably low in rural areas. Only 59% of women in rural areas had a birth attendant to help with delivery, compared to 90.2% in urban areas [10]. In Ghana, national guidelines recommend maternal and newborn care from pregnancy to the postnatal period. The government supports equitable access to such care through a national health insurance scheme. Nevertheless, in our previous study conducted in 2012, only 8% of women and their newborns received all recommended care [11].

In other low- and lower-middle-income countries, the previous interventions have been found effective in improving access to care for mothers and newborns [9]. However, most of these interventions took place within controlled environments; only a few have been conducted and examined in a real-world setting [12]. This gap between science and service has been demonstrated in several studies [12-14].

To overcome the gap, the Ghanaian and Japanese governments launched a joint project, the Ghana Ensure Mothers and Babies Regular Access to Care (EMBRACE) implementation study. This study is distinct because its aim was to demonstrate an intervention program’s effectiveness in a real-world setting, thereby facilitating its implementation on a national scale. Therefore, the study, which included over 21 000 women, examined the effectiveness of the intervention itself as well as the strategy used to implement it. The latter was undertaken to support the validity and sustainability of the intervention in a public health policy framework. The Reach Effectiveness Adoption Implementation Maintenance (RE-AIM) was adopted as the appropriate framework for assessing the strategy of implementation in this study. It was initially developed by Glasgow et al. for the consistent reporting of health research results [15], and it can also be used to assess the real-world impact of the intervention on individual and organizational levels using the following five factors: reach, effectiveness, adoption, implementation, and maintenance [12,15-17].

Therefore, this study evaluated the latter objective—the implementation process of the Ghana EMBRACE intervention program—using the RE-AIM framework in a real-world setting.

## METHODS

### Study design and setting

**Figure 1:**
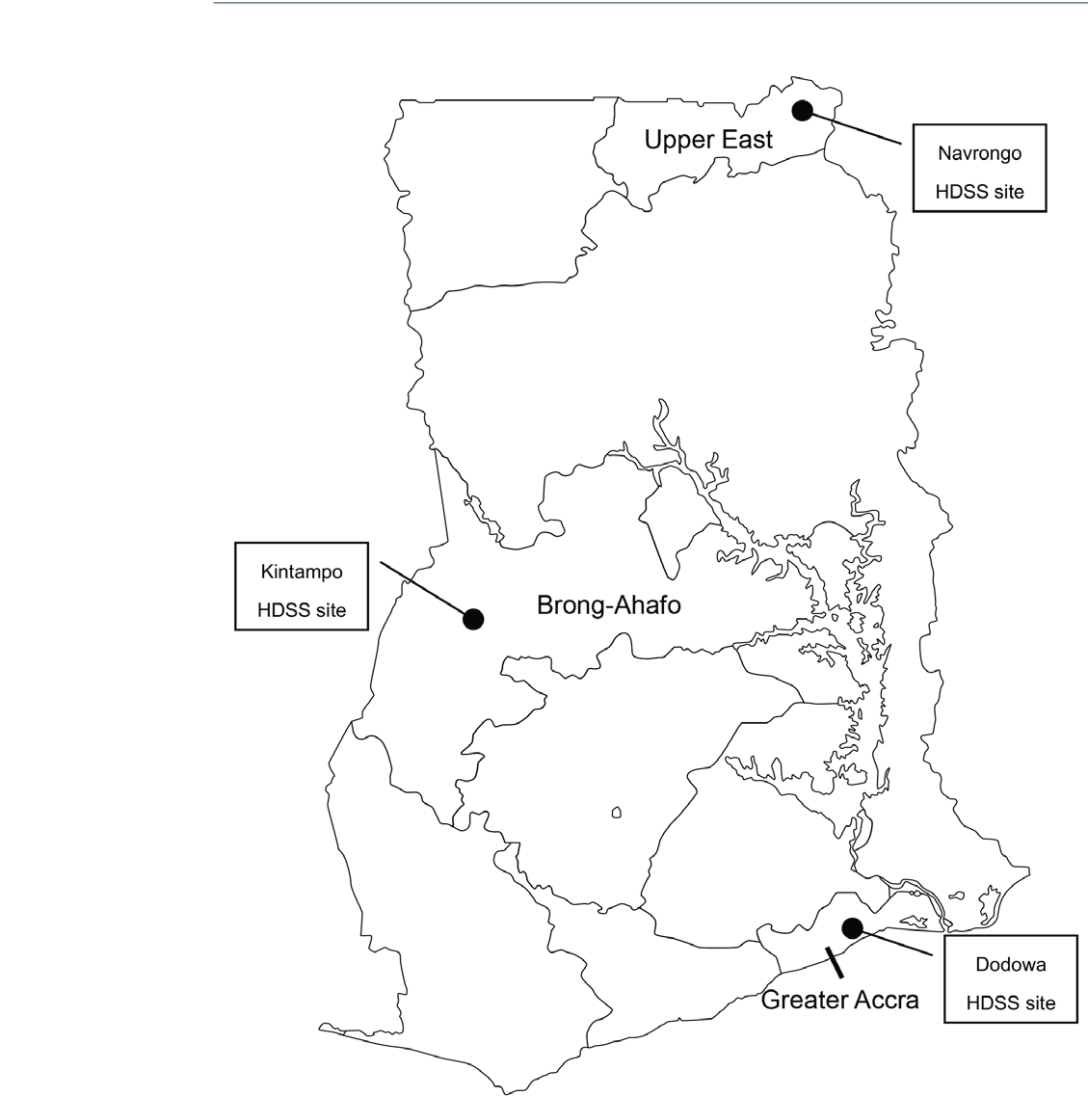
Study sites of Ghana EMBRACE Implementation Research.

The study design of the EMBRACE intervention was a cluster-randomized controlled trial based on an effectiveness-implementation hybrid design [18,19]. The intervention was conducted in Ghana for one year, from October 2014 to September 2015. In total, over 21 000 women participated in the EMBRACE intervention program with their newborns. In this study, we evaluated the effectiveness of the EMBRACE intervention program based on the interview results of randomly selected women and health workers as well as based on the intervention monitoring reports. Quantitative and qualitative data were collected using different approaches to evaluate the implementation process. The EMBRACE study details are in the protocol registered under the International Standard Randomized Controlled Trial Number (ISRCTN: 90618993) and in a published study protocol [20]. No significant amendment has been made in the methods since the trial commencement. The Ghana EMBRACE intervention program was conducted at the Health and Demographic Surveillance System (HDSS) sites in Dodowa, Kintampo, and Navrongo (**Figure 1**). These three sites represent the country's three ecological zones: coastal Savannah, forest belt, and northern Savannah. At each site, people’s basic medical histories have been continuously surveyed under the supervision of the Ghana Health Service.

Dodowa is in a rural, but rapidly urbanizing district with a total area of 1529 km [21]. The location of the households is spread over a larger field. Kintampo is located in the center of Ghana with a surface area of 7162 km [22]. Access to health facilities is challenging, and the home birth rate is higher here than in the other two sites. Navrongo lies in northern Ghana with an area of 1675 km [23]. Community-based health planning (CHPS), and the service program and community health officers (CHO) in Ghana were first established in Navrango.

### Randomization and allocation

**Figure 2:**
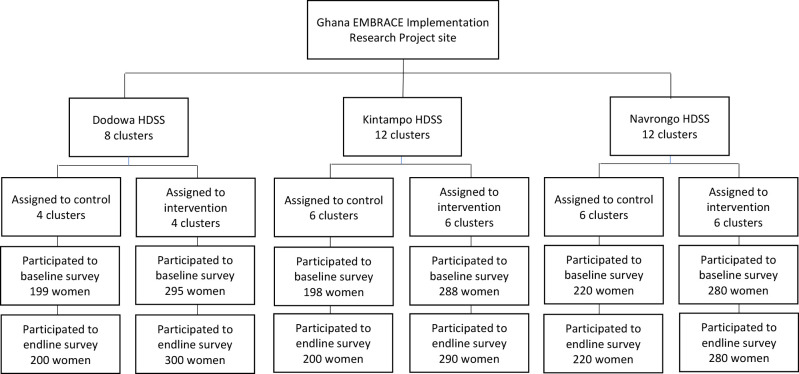
Participants in the implementation evaluation analysis.

In total, 32 sub-districts of the study site were randomly allocated to be intervention or control areas in a 1:1 ratio (**Figure 2**). In the intervention areas, 66 health facilities were included (three hospitals, eight private facilities, six health centers, and 49 CHPS zones/compounds); in the control area, 63 facilities were surveyed (one hospital, three private clinics, 10 health centers, and 49 CHPS zones/compounds). To conceal each area’s allocation, a data analyst, who was not a primary member of the study team, randomized the clusters using computer-generated random numbers. Due to the nature of the cluster-randomized controlled trial and intervention design, participants’ enrollment and intervention assignment were not concealed.

### Ghana EMBRACE intervention

The Ghana EMBRACE implementation research aimed to increase the CoC completion rate of mothers and newborns. The team developed an intervention program with three aims: reducing the coverage gap in PNC<48 hours postpartum, accelerating the understanding of the CoC among health care providers, and encouraging community members to support mothers in obtaining maternal and newborn care from skilled health workers.

Following Ghana’s national guidelines on maternal and child health, the CoC components in the Ghana EMBRACE intervention program were defined as four ANC visits; delivery attended by a skilled birth attendant; and PNC at 48 hours, 7 days, and 6 weeks postpartum. The program included four important components with unique codes for the various interventions [20]: 1) use of the CoC card (A-1), 2) CoC reorienting for health workers (A-2), 3) 24-hour retention of women and newborns at a health facility after delivery (B-1), and 4) PNC by home visits (B-2).

The CoC card (A-1) was a one-page pictorial educational and record card used by the health care providers to explain the value of CoC and encourage women to continuously receive care (Appendix S1 in the **Online Supplementary Document**). It was attached to the existing Maternal Health Record Book for easy reference. When a woman received services between 16 weeks of pregnancy and 6 weeks postpartum, her health worker placed a star sticker on her card to indicate her compliance with the CoC. A gold star indicated an on-time uptake, an orange star was for a delayed uptake, and no star denoted missed care. Trained District Health Management Team (DHMT) members provided an orientation to facilitate the understanding of the CoC (A-2) among health workers at the start of the intervention period. To increase the coverage of 48-hour PNC, mothers and newborns were retained at the health facility for 24 hours after delivery (B-1) and then provided PNC. The CHO, whose core mandate is to undertake home visits, visited mothers who delivered at home to provide PNC within 48 hours postpartum (B-2).

The program provided minimum materials such as a manual sphygmomanometer, stethoscopes, thermometers, and penlights for health checkups to all health facilities. In addition, the program provided beds to facilities to enable the provision of postpartum rest for the mothers and newborns under intervention B-1, and motorcycles to enable home visits to ensure completion of intervention B-2. The interventions were administered to women and their newborns through primary and secondary health facilities between 16 weeks of pregnancy and 6 weeks postpartum. The B-1 intervention was implemented only in Dodowa and Navrongo because fewer midwives were available in Kintampo. In the control area, no intervention was conducted, and standard maternal and newborn health care was provided as routine care at health facilities.

### Outcomes

This study targeted the implementation outcomes, assessed in terms of five criteria (reach, effectiveness, adoption, implementation, and maintenance) of the RE-AIM framework [12,15-17]. Details of each definition are presented in the analysis section.

### Participant sample

To evaluate the EMBRACE intervention, women recruited through random sampling were surveyed from the study site before (baseline survey) and after (endline survey) the intervention period. Participants in both baseline and endline surveys were women aged 15-49 years. The inclusion criterion was having given birth between 1 September 2012 and 30 June 2014 for the baseline survey, and between 1 October 2014 and 30 September 2015 for the endline survey. Exclusion criteria were declining to be interviewed or moving out of the target HDSS areas. The sample size for the surveys was set as 1500 (intervention: 750, control: 750) for each survey sample size, and the intra-class correlation coefficient (0.02675) was calculated based on the data collected from our formative study in the previous year at the same sites. A two-step cluster random sampling method was used. Geographical units were created in proportion to the cluster’s population size, and 50 geographical units were selected from each of the three HDSS sites. From within each geographical unit, 10 eligible women were randomly selected. The details of this process were presented in the study protocol [20].

The key informant interviews were also conducted with 18 health workers chosen through convenience sampling in the intervention arm. The intervention monitoring data were collected monthly from all 66 health facilities in the intervention area.

### Data collection

#### Questionnaire survey

A baseline and an endline survey were conducted in August 2014 and November 2015, respectively. The interviews were conducted by trained interviewers who visited the selected women’s homes. Using a structured questionnaire, women were asked about their sociodemographic characteristics, use of maternal and child health care services, complications experienced during pregnancy and till six weeks postpartum. Prior to the baseline and endline surveys, research assistants were trained at each HDSS site. Data were cross-checked with the maternal health record book, which each woman usually possessed.

#### Key informant interview

Key informant interviews were conducted with the health workers between November 2014 and November 2015 using an interview guide. The topics included changes in service quality, women’s responses to services, and the interviewees’ evaluation of the implementation fidelity and sustainability after the intervention. A member of the study team interviewed each health worker at the health facility for 30-45 minutes. The interviewer made notes or audio-recorded the interview.

#### Monitoring

The monitoring team collected intervention monitoring data at all health facilities of the intervention area for each month of the 12-month intervention period. The monitoring team was created at each HDSS site, comprising the persons in charge of supervision in the DHMT, Sub-District Health Management Team (SDHMT), and HDSS research members. Every month, the team visited all the health facilities located in the intervention areas for routine supervision to support the health service providers in implementing intervention programs in accordance with the study protocol. They also interviewed health workers and noted all the issues raised, good practices, requests, and mothers’ comments on the interventions; further, they noted the number of ANC or PNC check-up received during the monthly monitoring on a monitoring sheet.

### Analysis

In total, 34 components of the RE-AIM framework were reported [15,24] and classified under five general criteria of RE-AIM according to the operational definitions as listed in **Table 1**.

**Table 1:** RE-AIM criteria, definitions, and measurements

RE-AIM criteria	Definition*	Measurement
Reach	An individual-level measure of participation based on the percentage and characteristics of people who receive the intervention.	The total number and percentage of women who participated in each EMBRACE intervention component for both intervention and control areas.
Effectiveness	The impact of the intervention on important outcomes, including positive and negative effects and behavioral outcomes at the individual and institutional level.	The health outcomes (still birth, newborn’s death, complications) and health services received (ANC, delivery by skilled birth attendant, PNC, CoC completion) of women and newborns, comparing data of intervention and control areas.
Adoption	The proportion and representativeness of settings and intervention of agents who are willing to implement a program.	The number and proportion of health facilities and women who adopted the intervention program.
Implementation	The extent to which an intervention is delivered as intended.	Qualitative assessment of the extent to which the intervention is delivered as intended and the implementation cost.
Maintenance	The extent to which a program becomes institutionalized at the organization level. It is also applied at the individual level by evaluating the long-term effects of the program on the outcomes.	The extent to which a program becomes institutionalized at the organization level.

EMBRACE – Ensure Mothers and Babies Regular Access to Care, ANC – antenatal care, PNC – postnatal care, CoC – continuum of care

*Definitions of RE-AIM five criteria: Glasgow et al., 1999 [15].

Survey data were analyzed descriptively to compare the distribution of women’s characteristics and program coverage. Bivariate analyses were performed to compare differences between intervention and control areas using the *t* test and the χ^2^ test. Relative risks of maternal/newborn complications and stillbirth/neonatal deaths were assessed for the intervention area relative to the control area. Statistical significance was set at *P* < 0.05. These data were analyzed using an intention-to-treat analysis, with SPSS version 25 (IBM Co., Armonk, NY, USA).

Coding was performed for the scripts of the audio-recorded data from key informant interviews, interview notes, and intervention monitoring reports. The codes were then analyzed using content analysis according to the categories of the related RE-AIM elements.

### Ethics

Ethical approval was obtained from the Ethics Review Committee of the Ghana Health Service (reference: GHS-ERC: 13/03/14); the institutional review boards of Dodowa HRC (reference: FGS-DHRC: 280214), Kintampo HRC (reference: 2014-11), and Navrongo HRC (reference NHRCIRB137) in Ghana; and the Research Ethics Committee of the Graduate School of Medicine of the University of Tokyo in Japan (reference serial number: 10513). In addition, we collected informed consent from all the study participants before including them in the study. All study procedures were performed in accordance with the principles included in the Declaration of Helsinki.

## RESULTS

The number of women who participated in the baseline and endline surveys was 1480 (intervention: 863 vs control: 617) and 1490 (intervention: 870 vs control: 620), respectively. The other complete data set included the notes and audio-recorded data from 18 key informant interviews: eight midwives and 10 CHOs/community health nurses (CHNs). Twelve sets of intervention monitoring reports were collected and analyzed from each one of the 66 health facilities.

**Table 2** provides a detailed profile of the women living in the study site. While sociodemographic characteristics of those living in the intervention and control areas were similar, the wealth quintiles (*P* < 0.01) and travel time to a facility (*P* = 0.08) were statistically different at the baseline.

**Table 2:** Characteristics of Ghana EMBRACE Implementation Research Intervention

Characteristics	Baseline survey						Endline survey				
**Intervention (n = 863)**		**Control (n = 617)**		***P*-value**		**Intervention (n = 870)**		**Control (n = 620)**		***P*-value**
**n**	**%**	**n**	**%**			**n**	**%**	**n**	**%**	
**Maternal characteristics**											
Living area					0.23						0.20
Dodowa	295	34.2	199	32.2			300	34.5	200	32.3	
Kintampo	288	33.4	198	32.1			290	33.3	200	32.3	
Navrongo	280	32.4	220	35.7			280	32.2	220	35.5	
Age (years):					0.90						0.80
≤20	53	6.1	35	5.7			130	14.9	92	14.8	
20-29	448	51.9	317	51.4			470	54.0	332	53.5	
30-39	301	34.9	223	36.1			239	27.5	166	26.8	
≥40	61	7.1	42	6.8			31	3.6	30	4.8	
Education level:					0.78						0.31
None	257	29.8	178	28.8			182	20.9	145	23.4	
Primary	222	25.7	170	27.6			242	27.8	196	31.6	
Middle	289	33.5	209	33.9			326	37.5	207	33.4	
Secondary and above	95	13.0	60	9.7			120	13.7	72	11.6	
Marital status:					0.12						0.32
Married	542	62.8	415	67.3			470	54.0	351	56.6	
Cohabiting	224	26.0	150	24.3			260	29.9	163	26.3	
Divorced/separated/widowed/never married	97	11.2	52	8.4			140	16.1	106	17.1	
**Family characteristics**											
Age of partner (years, baseline = 1269, endline = 1234):*						0.19					0.76
≤29	168	22.8	146	23.7			219	30.2	165	26.6	
30-39	312	42.3	231	37.4			333	45.9	223	36.0	
40-49	190	25.7	121	19.6			136	18.8	99	16.0	
≥50	68	9.2	33	5.3			37	5.1	22	3.5	
Education level of partner (baseline = 1272, endline = 1223):*						0.14					0.01
None	198	26.9	144	23.3			142	16.3	128	20.6	
Primary	118	16.0	94	15.2			114	13.1	87	14.0	
Secondary	214	29.1	191	31.0			243	27.9	167	26.9	
Tertiary and above	206	28.0	107	17.3			218	25.1	124	20.0	
Wealth quintiles						<0.01					<0.01
Highest	187	21.7	108	17.5			204	23.4	93	15.0	
Higher	169	19.6	120	19.4			192	22.1	106	17.1	
Middle	196	22.7	104	16.9			174	20.0	118	19.0	
Lower	155	18.0	141	22.9			112	12.9	132	21.3	
Lowest	156	18.1	144	23.3			188	21.6	171	27.6	
Travel time to antenatal care facility (minutes; baseline = 1381, endline = 1479)*						0.08					<0.01
≤30	509	63.9	339	54.9			594	68.8	369	59.5	
31-60	220	27.5	166	26.9			207	24.0	180	29.0	
61-90	22	2.7	41	6.6			28	3.2	22	3.5	
≥91	50	6.2	34	5.5			35	4.1	44	7.1	
Have a national health insurance card:											
Yes	510	59.1	344	55.8	0.21		611	70.2	407	65.6	0.06

*Those with an unknown status were excluded.

### Evaluation according to RE-AIM framework

Details of our evaluation of the EMBRACE intervention program according to the RE-AIM framework, are presented in Appendix S2 in the **Online Supplementary Document** along with the 34 RE-AIM assessment items.

#### Reach

As shown in **Table 3****,** 72.8% of the women who participated in the endline survey in the intervention area had a CoC card. Although the card was not distributed in the control area, 21.6% of endline survey respondents in that area had one. In the intervention area, the percentage of women who stayed at a facility for at least 24 hours postpartum (44.5% vs 53.7%), and the percentage of women who received home-visit PNC within 48 hours (2.9% vs 14.0%) increased between the pre- and post-intervention periods. The increase in the rate of women receiving these services was observed in the control area as well, even though the research team did not implement any interventions there.

**Table 3:** Health care and intervention coverage and health outcomes

CoC characteristics	Baseline survey					Endline survey					Relative risk
**Intervention (n = 863)**		**Control (n = 617)**		***P*-value**	**Intervention (n = 870)**		**Control (n = 620)**		***P*-value**	
**n**	**%**	**n**	**%**		**n**	**%**	**n**	**%**		**(95% CI)**
EMBRACE intervention involvement											
Received the CoC card	–	–	–	–	–	632	72.8	134	21.6	<0.01	
Stayed for 24h at health facility after delivery	384	44.5	276	44.7	0.93	467	53.7	320	51.6	0.43	
Received home visit postnatal care within 48h*	7	2 · 9	6	3 · 6	–	21	14.0	15	12.6	0.74	
Received any of the above components†	–	–	–	–	–	736	84.6	396	63.9	<0.01	
Refused to stay for 24h at health facility after delivery‡	57	14.4	41	13.7		38	7.6	21	6.3		
Antenatal care:											
4 or more visits	590	68.4	415	67.3	0.65	669	76.9	479	77.3	0.87	
Delivery:											
Delivery by the skilled birth attendant	631	73.1	463	75.0	0.41	713	82.0	496	80 · 0	0.34	
Postnatal care:											
Received within 48h	465	53.9	321	52.0	0.49	706	81.5	498	80.3	0.74	
Received around 1 week (3 - 7 d)	274	31.7	180	29.2	0.26	637	76.8	425	68.5	0.04	
Received around 6 weeks (8 - 48 d)	405	46.9	314	50.9	0.10	667	79.0	452	68.5	0.07	
Completed continuum of care	65	7.5	57	9.2	0.24	410	47.1	246	39.7	<0.01	
Stillbirth/postnatal death within 48 d	11	1.3	11	1.8	0.43	18	2.1	10	1.6	0.52	1.17 (0.71-1.93)
Complications among women:§											
During pregnancy	309	35.8	217	35.2	0.80	322	37.0	235	37.9	0.73	0.98 (0.86-1.11)
During delivery	120	13.9	97	15.7	0,33	111	12.8	85	13.7	0.59	0.95 (0.80-1.13)
At 6 weeks after delivery	96	11.2	82	13.4	0.20	68	7.9	50	8.1	0.88	0.98 (0.79-1.22)
Complications among newborns:‖											
Danger signs immediately after birth	77	8.9	58	9.4	0.75	61	7.0	61	9.8	0.05	0.82 (0.68-0.99)
At 6 weeks after birth	131	15.3	125	20.4	0.01	104	12.2	97	15.8	0.05	0.85 (0.72-0.99)

CoC – continuum of care, CI – confidence interval

*Denominator is the numbers of women who delivered at own home or TBA's home. Baseline (intervention = 243, control = 165), Endline (intervention = 150, control = 119).

†Questions asked only during the baseline survey.

‡Denominator is the number of women advised by the health workers to stay in the facility for 24 h after delivery. Baseline (intervention = 396, control = 299), Endline (intervention = 499, control = 334).

§Complications among women include: (during pregnancy) abdominal pains, swellings, excessive vomiting, severe headache, anemia, bleeding; (during delivery) preterm birth, heavy bleeding, premature rupture of the membrane, prolonged delivery; (at 6 weeks after delivery) fever, easily felt tired, a problem in urination, breast problem.

‖Complications among newborns include: (danger signs immediately after birth) very small, difficulty in breathing, fever, cold body, bleeding spots, too weak to suck, did not cry; (at 6 weeks after birth) fever, diarrhea, vomiting every feed, skin pustules or boils, excess crying, cough, not gaining weight, not feeding well, jaundice, cold body.

#### Effectiveness

As shown in **Table 3**, the proportion of women who had completed CoC was not statistically different between intervention and control groups in the baseline survey (7.5% vs 9.2%, *P* = 0.24). However, the proportion increased at the endline survey and was significantly different (47.1% vs 39.7%, *P* < 0.01). The proportion of newborns with danger signs immediately after birth was not statistically different between intervention and control groups at the baseline survey (8.9% vs 9.4%, *P* = 0.75). However, the proportion decreased in the intervention group and was significantly different from that of the control group at the endline survey (7.0% vs 9.8%, *P* = 0.05) (relative risk (RR) = 0.82, 95% confidence interval (CI) = 0.68-0.99]). The proportion of newborns who had complications at six weeks postpartum was statistically different between intervention and control groups at the baseline survey (15.3% vs 20.4%, *P* = 0.01). These proportions were different at the endline survey as well, with a smaller proportion of newborns having complications in the intervention areas (12.2% vs 15.8%, *P* = 0.05) (RR = 0.85, 95% CI = 0.72-0.99).

In the key informant interviews, health workers said that newborn complications were detected early because of the home visit PNC intervention: the effect of the intervention program on the early detection of complications among newborns. Regarding the intervention promoting a 24-hour stay at the birth facility, one health worker reported that she could detect newborn’s fever because the mother stayed overnight at the facility. During home-visit PNC, another health worker found inappropriate local practices that required proper instruction:

“By visiting home, many cases of neonatal sepsis could be detected. This is due to bad umbilical cord cutting practices, such as the use of salty sand and toothpaste.” (CHO)

#### Adoption

**Figure 3:**
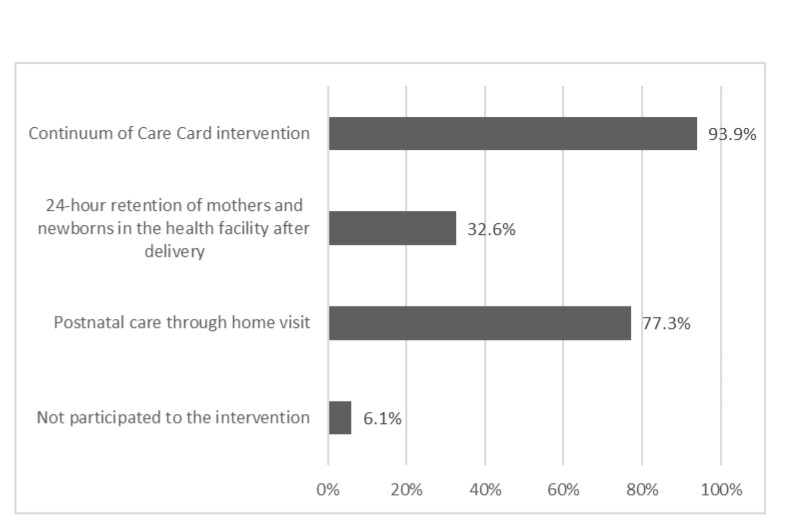
Percentage of health facilities that adopted EMBRACE program according to the intervention types (n = 66).

**Figure 3** shows the percentage of health facilities that were able to provide EMBRACE interventions to women and newborns from the perspective of personnel and infrastructure capacity. Among the 66 health facilities in the intervention areas, CoC cards were used in 93.9% of the facilities. Excluding Kintampo, where access to delivery facilities is limited, 32.6% of the health facilities participated in the intervention promoting a 24-hour stay in the birth facility. The absence of a midwife and lack of a bathroom were the main reasons for nonparticipation. Home visits were implemented in 77.3% of the health facilities. Among all the health facilities, 6.1% did not implement any of the intervention components because they had fewer health workers than the other facilities.

In the intervention area, 87.2% (n = 552) of the women who received the CoC card indicated a willingness to re-use the card in subsequent pregnancies (not stated in the table). The proportion of women who refused to stay in the birth facility for 24 hours postpartum decreased from 14.4% to 7.6% in the intervention areas between pre- and post-intervention. However, it also decreased in the control area, from 13.7% to 6.3%.

The key informants had relatively positive assessments of the women’s acceptance of the interventions. Particularly, PNC home visits were highly accepted as many women are unable to receive PNC at a health facility within 48 hours of birth due to cultural barriers. Thus, home visits are the only way for them to receive PNC.

“Traditionally, women of this region do not go out for 40 days after delivery. So, it is good to visit their homes and see whether the mother and baby are fine.” (CHO)

In contrast, health care workers expressed both positive and negative opinions of the intervention. Positive opinions were related to lower workloads than before due to the regularization of pregnant women's facility visits. Since the timing of care was clearly communicated via the CoC card, the number of unexpected visits by pregnant women and the long queues for examinations were reduced. However, home-visit PNC was considered a challenge for health workers in charge of areas where households were dispersed and required long-distance travel.

#### Implementation

The monitoring reports indicated several implementation challenges in the beginning (Appendix S3 in the **Online Supplementary Document**). The main obstacles preventing the CoC card intervention included cards being out of stock and misunderstood card usage. Besides, those health workers who had attended the CoC orientation did not pass on the knowledge and skills they had acquired to the new staff members after staff turnover. The intervention promoting a 24-hour stay was not provided at certain health facilities due to the renovation of wards or the lack of sanitary facilities. The home-visit PNC intervention was initially challenged because, at the beginning of the intervention period, health workers had difficulty riding the motorcycles. This challenge was resolved through on-the-job guidance from EMBRACE study monitoring members and supervisors of the DHMT. When women and their newborns did not stay in the facility for 24 hours after delivery, health workers followed up with home-visit PNC arranged by phone or group chat application.

The estimated implementation cost of the intervention beyond the surveys, equipment, and research meetings was 344 000 USD per year; a CoC card's charge was only 0.5 USD each. The most expensive aspect of the implementation process was the initial training and monitoring, which translated to approximately 21 USD per person/y, as calculated based on the number of women who received CoC cards during the one-year intervention period. The procured equipment cost 244 000 USD. The baseline and endline surveys for the intervention and control groups altogether cost 328 000 USD. Other costs were mostly related to research meetings (facility utilization for three sites, monthly monitoring, and the three workshops organized). They amounted to 217 000 USD per year. There were no implementation costs associated with the control area since the intervention was not implemented there.

#### Maintenance

After the EMBRACE intervention program had been implemented, Ghanaian and Japanese governments collaborated to integrate the maternal and child health record books into a single Maternal and Child Health Record Book, which combines all records from ANC, delivery care, PNC, and child health visits according to CoC principles. Since March 2018, the new book has gradually been replacing the former books in Ghana. The new record book also includes the CoC card as one of its pages to encourage women to access and receive necessary care. This new card stipulates two major care-related changes: 1) the number of ANC visits has been increased from four to eight, and 2) children’s care has been extended till they are 24 months of age. Instead of stickers, an ink stamp is placed on the card at each care visit.

## DISCUSSION

Although more than 70 studies have used the RE-AIM framework [24], our study is unique as it evaluated the RE-AIM items in detail and obtained several important findings. More than 85% of women in the intervention area participated in the EMBRACE intervention (Reach). Mothers in the intervention area were more likely to maintain CoC while completing the recommended series of health care visits and were less likely to experience newborn complications (Effectiveness). In the intervention area, approximately 94% of health facilities participated in the EMBRACE intervention (Adoption). Although certain logistic and personnel challenges were identified at the beginning of the intervention, these were resolved by the monitoring members and district supervisors (Implementation). The CoC concept was introduced into Ghana’s routine public health program, and the responsible agency developed a new Maternal and Child Health Record Book (Maintenance).

The EMBRACE was one of those rare studies that evaluated both an intervention as well as its implementation using the effectiveness-implementation hybrid design. This design enabled our study to reduce the science-to-service gap [18] by presenting findings intended to incorporate real-world public health policy. The obstacles and catalysts affecting the implementation process were identified multi-directionally, and were not usually evaluated in the ordinal impact evaluation design of randomized controlled trials. The advantages of this study design have been pointed out in a previous study [18]. They have been effectively demonstrated in our study.

The RE-AIM-based evaluation identified the strong potential for nationwide expansion of the program. Mainly, the intervention's adoption was relatively high as most of the health facilities in the intervention area participated in the Ghana EMBRACE intervention program. Only in 6% of cases did the facilities hardly provide any health care. The high adoption rate might be due to the flexibility afforded to the facilities in choosing the intervention components that worked for them. The health care facilities had to provide the CoC card but could choose to offer either the 24-hour stay, or the home-visit PNC, or both. The interventions were usually not equally applicable to all contexts. They required flexible application, considering the reality of the individuals and organizations involved. This implementation policy can be matched to resource-limited settings. It should be considered when the intervention program is expanded for use at the national level.

This study linked research outputs to a real public health policy. Its effectiveness contributed to the policymakers’ decision to integrate the study into a real-world approach. In particular, the proportion of mothers who completed CoC vastly increased through the intervention. Furthermore, complication risks among newborns decreased both immediately and six weeks after birth. The CoC card could be one of the contributors to the increase in CoC, as our findings showed that women received subsequent care after using the CoC card [25]. As a whole, the intervention program indicated the effectiveness of improving the availability of care in the most critical period for a newborn by boosting the newborns’ health status.

The EMBRACE intervention can be feasible in other settings with limited resources as well. The intervention's basic concept aims at universal application, and the intervention is simple enough to be implemented in different locations using affordable resources. Thus, it can be used in other low- and lower-middle-income countries in sub-Saharan Africa or South Asia where the CoC needs to be improved.

This study had several limitations. First, a lack of human resources and infrastructure problems in health facilities were faced during the implementation process, possibly affecting the intervention's generalizability. Second, some women of the control area received some elements of the EMBRACE intervention program because they were free to change a health facility anytime during pregnancy, such as CoC cards, which may have made it harder to determine the program’s effectiveness by clouding the differences between the intervention and control groups. However, this can also be considered as a sign of the program’s strength. It appears that the reputation of our interventions spread naturally by word of mouth; hence, CoC cards or interventions similar to ours were undertaken by others without any extra cost to the program. Third, the provision of equipment might alter generalizability in real-world settings. Fourth, the findings were identified relatively early after the end of the intervention period. Long-term effects, such as mortality rates and sustainability of the intervention, were not assessed in this study.

## CONCLUSIONS

Despite such limitations, this study demonstrated the importance of evaluating implementations initiated in research. Furthermore, it contributed vitally in assessing the adoption of the EMBRACE intervention in a real-world setting and accelerating the program for nationwide scaling across. In addition to its national application in Ghana, the program is expected to be used in pilot trials in other low- and lower-middle-income countries.

## Additional material

Online Supplementary Document
