# Supplementary material for: EMBRACE intervention to improve the continuum of care in maternal and newborn health in Ghana: The RE-AIM framework-based evaluation
Source: J Glob Health. 2021 Mar 27;11:04017. doi: 10.7189/jogh.11.04017 (PMC8005313; doi:10.7189/jogh.11.04017)
Supplement: Online Supplementary Document [file jogh-11-04017-s001.pdf]

Appendix S1. Continuum of Care Card

Continuum of Care Card

Health facility & ANC No.:

Name:

CoC SERVICES

ANC1 by 16 weeks (by 4 months)	ANC2 24-28 weeks (6-7 months)	ANC3 at 32 weeks (at 8 months)	ANC4 at 36 weeks (at 9 months)	Skilled delivery Facility delivery	PNC1 by 48hours (by 2 days)	PNC2 at 7 days	PNC3 at 6 weeks
Date at 16 weeks	Date at 28 weeks	Date at 32 weeks	Date at 36 weeks	Expected delivery date	Date at 2 days	Date at 7 days	Date at 6 weeks
Date of actual visit	Date of actual visit	Date of actual visit	Date of actual visit	Date of actual visit	Date of actual visit	Date of actual visit	Date of actual visit
Mother ☆	Mother ☆	Mother ☆	Mother ☆	Delivery ☆	Mother ☆	Mother ☆	Mother ☆
					Baby ☆	Baby ☆	Baby ☆

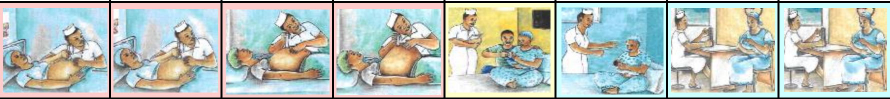

ESSENTIAL SERVICES

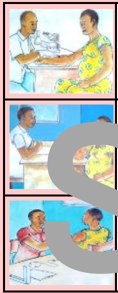

	ANC1	ANC2	ANC3
Blood test	Blood test 1 (Hb 1) ☆		Blood test 2 (Hb 2) ☆
Malaria	Malaria Drug 1 (IPT1) ☆	Malaria Drug 2 (IPT2) ☆	Malaria Drug 3 (IPT3) ☆
Tetanus	[illegible]anus Toxoid [illegible]on 1 ☆	[illegible]etan[illegible]oxoid [illegible] ☆	

Sample

Blood group
Rhesus factor ☆

HEALTH EDUCATION

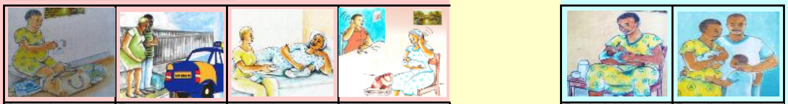

Items for Delivery & Baby ☆	Transportation for delivery ☆	Caregiver ☆	Call care provider after delivery ☆	Early initiate, Exclusive Breastfeeding ☆	Family planning ☆

DANGER SIGNS

If Yes, see the detail record

ANC1	ANC2	ANC3	ANC4	Delivery	PNC1	PNC2	PNC3
Mother ☐ YES ☐ NO	Mother ☐ YES ☐ NO	Mother ☐ YES ☐ NO	Mother ☐ YES ☐ NO	Mother ☐ YES ☐ NO	Mother ☐ YES ☐ NO	Mother ☐ YES ☐ NO	Mother ☐ YES ☐ NO
				Baby ☐ YES ☐ NO	Baby ☐ YES ☐ NO	Baby ☐ YES ☐ NO	Baby ☐ YES ☐ NO

Contact number of Health care provider

✂ CUT HERE BY HEATH CARE PROVIDER AT 6 WEEK AFTER DELIVERY

ANC1	ANC2	ANC3	ANC4	Delivery	PNC1	PNC2	PNC3
☆	☆	☆	☆	☆	☆	☆	☆
☆	☆	☆	☆	☆	☆	☆	☆
					☆	☆	☆
					☆	☆	☆

Health faciltiy & ANC numberr Name

Appendix S2. RE-AIM Framework Criteria and Assessment

	Criterion	Assessment
**Reach**		
1.	Exclusion criteria (% excluded or characteristics)	Women were excluded if they refused to participate, declined to be interviewed, or had moved out of the target sites.
2.	Percentage of individuals who participated, based on valid denominator	Percentage of women who received the intervention components in the intervention and control areas were: CoC card: 72.8% vs. 21.6%. Percentage of women who had stayed at a facility for at least 24 hours postpartum (44.5% vs. 53.7%), and the percentage of women who had received home-visit PNC within 48 hours (2.9% vs. 14.0%), increased between the pre- and post-intervention periods.
3.	Characteristics of participants compared with nonparticipants	Most of the sociodemographic characteristics of participants did not differ significantly between the intervention and control groups. However, the wealth quintiles and travel time to health facilities were differed.
4.	Use of qualitative methods to understand recruitment	Summarized in the intervention procedure report.
**Effectiveness**		
5.	Measure of primary outcome	Continuum of care completion rate: increased from 7.5% to 52.9% in the intervention area and from 9.2% to 39.7% in the control area. Relative risk of neonatal complication immediately after birth and at 6 weeks after birth were 0.82 (0.68-0.99) and 085 (0.72-0.99), respectively.
6.	Measure of primary outcome relative to public health goal	No national data was available regarding the rate of continuum of care completion and neonatal complication. Coverage rates of antenatal care 4 times or above and delivery by skilled birth attendants were 76.9% and 82.0% respectively after intervention. Antenatal care rate was lower than the national survey, 87.3% in 2014 [1]. The skilled birth attendance rate was higher than the national survey, 55.3% in 2013 [2].
7.	Measure of broader outcomes or use of multiple criteria (e.g. quality of life or potential negative outcome)	Multiple outcomes assessed, but quality of life. No evidence of negative outcomes.
8.	Measure of robustness across subgroups (e.g. moderation analysis)	Difference in outcomes between intervention sites.
9.	Measure of short-term attrition (%) and differential rates by treatment group	Rate differential between intervention sites.
10.	Use of qualitative methods to understand outcomes	Summarized in effectiveness evaluation report.
**Adoption**		
**Adoption: setting level**		
11.	Setting exclusion (% or reasons or both)	6% of facility did not participated to intervention due to lack of health personnel.
12.	Percentage of settings approached that participated (valid denominator)	No refusals of 66 approached.
13.	Characteristics of settings participating (both comparison and intervention compared with either nonparticipants or some relevant resource data)	Summarized in study setting and appendix 1 including population, maternal demographic information, and regional characteristics.
14.	Use of qualitative methods to understand setting level adoption	Summarized in adoption evaluation report.
**Adoption: staff level**		
15.	Staff exclusions (% or reasons or both)	None excluded.
16.	Percent of staff offered that participate	All maternal health related staff participated including midwives and nurses.
17.	Characteristics of staff participants v nonparticipating staff or typical staff	No comparison has been done because all staff participated.
18.	Use of qualitative methods to understand staff participation or staff level adoption	Summarized in adoption evaluation report.
**Implementation**		
19.	Percent of perfect delivery or calls completed (e.g. fidelity)	Summarized in Table 2.
20.	Adaptations made to intervention during study (not fidelity)	Summarized in appendix 4. Challenges were identified at the first month of the intervention and relevant measures were taken.
21.	Cost of intervention: time	Two follow-up training for personnel change.
22.	Cost of intervention: money	Change for damaged materials and consumables: low cost
23.	Consistency of implementation across staff, time, settings, subgroups	Routines specified, monitored, and fed back in quality improvement cycle.
24.	Use of qualitative methods to understand implementation	Summarized in implementation evaluation report.
**Maintenance**		
**Maintenance: individual**		
25.	Measure or primary outcome (with comparison with a public health goal) at >=6 months follow-up after final treatment contact	No available data regarding >=6 months follow-up after final treatment contact.
26.	Measure of primary outcome at >=6 months	Continued collection of demographic and health surveillance of each

	follow-up after final treatment contact	study site.
27.	Measure of broader outcome (e.g. measure of quality of life or potential negative outcome) or use of multiple criteria at follow-up	Continued collection of demographic and health surveillance of each study site.
28.	Robustness data: something about subgroup effects over the long-term	Continued collection of demographic and health surveillance of each study site.
29.	Measure of long-term attrition (%) and differential rates by patient characteristics or treatment condition	Continued collection of demographic and health surveillance of each study site.
30.	Use of qualitative methods data to understand long-term effects	Continued collection of demographic and health surveillance of each study site.
**Maintenance: setting**		
31.	If program is still ongoing at>=6 months post-treatment follow-up	The intervention was piloted for 6 months in another regions of Ghana: Ashanti, Upper West, and Central.
32.	If and how program was adopted long-term (which elements retained after program completion)	One part of the intervention (CoC card) was be adopted in the new Maternal and Child Health Record Book.
33.	Some measure or discussion of alignment to organization mission or sustainability of business model	Discussion of including CoC completion rate into one of the indicators of the demographic and health surveillance system.
34.	Use of qualitative methods data to understand setting level institutionalization	CoC card was institutionalized into the Maternal and Child Health Record Book.

1. Ghana statistical service, Ghana health service, international. I. Ghana demographic and health survey 2014. MD USA: 2015.

2. Ministry of Health Ghana. Holistic assessment of the health sector programme of work 2013. Accra, Ghana: 2014.

Appendix S3. Challenges identified at the first month of the intervention and relevant measures during continuous monthly monitoring.

Intervention type	Challenges to implementation	Relevant measure
Use of the CoC card (A-1)	- CoC cards were out of stock in health facilities.<br>- CHOs provided CoC cards twice to the same mother.<br>- CHOs did not provide CoCs to mothers due to some misunderstanding.<br>- CHOs did not fill CoC cards correctly.	- The research team developed a job aid for a logistical communication system to avoid logistics problems.<br>- Supervisors of the DHMT and SDHMT administered instructions to CHOs how to use the CoC card during supervision.
CoC reorientation for health workers (A-2)	- Knowledge and skill acquired during the orientation were not taken over to the incoming health workers.	- The DHMT and the research team provided training to newly assigned health workers.
24-hour retention of women and newborns at a health facility after delivery (B-1)	- Mothers could not stay for 24 hours after delivery due to the renovation of the maternity ward.<br>- Mothers did not stay for 24 hours after delivery due to lack of a bathroom.	- CHOs followed up postnatal care of mothers and babies with a home visit. Group message application of smartphone (Whatsapp©) was used to send messages from health facility staff to the CHOs of the community to determine the follow-up postnatal care of the mother.
Postnatal care by home visits (B-2)	- CHOs had trouble riding motorbikes and were unable to visit mothers.	- The DHMT and SDHMT provided motorbike training to CHOs.<br>- CHOs asked volunteers to ride.

CoC: Continuum of Care, CHO: Community Health Officer, DHMT: District Health Management Team, SDHMT: Sub-District Health Management Team.
